# Marine Sponge Lectins: Actual Status on Properties and Biological Activities

**DOI:** 10.3390/molecules20010348

**Published:** 2014-12-26

**Authors:** Sandro Mascena Gomes Filho, Juscélio Donizete Cardoso, Katya Anaya, Edilza Silva do Nascimento, José Thalles Jucelino Gomes de Lacerda, Roberto Mioso, Tatiane Santi Gadelha, Carlos Alberto de Almeida Gadelha

**Affiliations:** 1 Laboratório de Proteômica Estrutural, Departamento de Biologia Molecular, Universidade Federal da Paraíba, Cidade Universitária, João Pessoa, PB CEP 58059-900, Brazil; 2 Faculdade de Ciências da Saúde do Trairi, Universidade Federal do Rio Grande do Norte, Centro, Santa Cruz, RN CEP 5900000, Brazil; 3 Núcleo de Engenharia de Pesca, Universidade Federal de Sergipe, Avenida Marechal Rondon, s/nº, São Cristóvão, SE CEP 49100000, Brazil

**Keywords:** marine sponge, lectins, properties, biological activities

## Abstract

Marine sponges are primitive metazoans that produce a wide variety of molecules that protect them against predators. In studies that search for bioactive molecules, these marine invertebrates stand out as promising sources of new biologically-active molecules, many of which are still unknown or little studied; thus being an unexplored biotechnological resource of high added value. Among these molecules, lectins are proteins that reversibly bind to carbohydrates without modifying them. In this review, various structural features and biological activities of lectins derived from marine sponges so far described in the scientific literature are discussed. From the results found in the literature, it could be concluded that lectins derived from marine sponges are structurally diverse proteins with great potential for application in the production of biopharmaceuticals, especially as antibacterial and antitumor agents.

## 1. Introduction

Marine sponges are primitive metazoans capable of producing a large amount of compounds that protect them both from undesirable predators as well as from infection by other pathogens. They are considered true chemical factories by producing hundreds of unusual compounds, many of which could be isolated and their structures elucidated, although their biological activity is still unknown [1,2]. Due to their prevalence, their distribution and ability to synthesize a large variety of compounds, marine sponges are promising organisms for the isolation of new bioactive molecules as potential sources of new drugs for the treatment of various diseases [3,4]. One example was given in 1999, when researchers isolated C-nucleoside from *Cryptotethya crypta*, which provided the chemical bases of cytarabine, the first anticancer drug derived from a marine source, whose clinical use for the treatment of leukemias was subsequently approved [5].

Lectins are proteins or glycoproteins that recognize carbohydrates widely distributed in nature. The recognition occurs through a carbohydrate recognizing domain (CRD) without carbohydrate modification, since lectins are not enzymes [6,7]. Although they are ubiquitous proteins, the lectins most studied to date are those belonging to the Leguminosae family, which includes plants, such as *Phaseolus* spp., *Vigna* spp. and *Canavalia* spp. [8]. Compared to the vegetable kingdom, few phyla have been studied in the animal kingdom for the presence and distribution of lectins. In the specific case of invertebrates, very few lectins have been isolated and compared to the many different plant lectins already isolated. The first description of lectin from marine sponge was conducted by Dodd* et al.* [9]. Later, Mebs and colleagues evaluated the hemagglutination capacity of 48 sponges collected in the Red Sea, in the Barrier Reef of Australia and the Florida Keys. The results showed that 42% of species studied had lectins capable of hemagglutinating human erythrocytes [10]. More than a decade later, 22 species of 12 families of tropical sponges were analyzed, and the hemagglutination activity was found in 10 of the species tested. Species that stood out due to their strong hemagglutination activity were from *Aplysina* spp. [11].

Like other natural products isolated from marine organisms, lectins and other proteins from marine sponges have shown great potential as candidates for new drugs, due to their wide range of biological activity, such as antibacterial, pro-inflammatory and even antitumoral [12,13]. Given the diversity of molecules that marine sponges are capable of producing and the scarcity of literature data, this review is mainly aimed at describing the current state of research on lectins isolated from marine sponges, highlighting their properties and biological activities. 

## 2. Characteristics of Lectins from Marine Sponges 

Currently, it is accepted that for a protein to be classified as a lectin, it must have the following features: present a carbohydrate recognizing domain (CRD), not have immune origin and, finally, not have catalytic activity [14,15,16]. In marine sponges, all lectins already studied meet these requirements. Moreover, many of them are glycoproteins with carbohydrate amounts ranging from 0.5% to 27.6% [11,17]. In relation to how the subunits of these lectins are organized, there is a mix of tertiary and quaternary arrangements ranging from monomers to octamers [11,17,18,19,20]. Table 1 summarizes the main characteristics of lectins from the marine sponges studied to date.

**Table 1:** Summary of the main characteristics of lectins from marine sponges.

Marine Sponges	Molecular Weight (kDa)	Conformation	Disulfide Bonds	Thermal Stability	Specificity	Carbohydrates (%)	Author
*Aplysina archeri*	16	Tetramer	−	N. D.	β-d-Gal-Oligossaccharides	3.5	[11]
*Aplysina lawnosa*	16	Tetramer	−	N.D.	β-d-Gal-Oligossaccharides	5	[11]
*Axinella polypoides I*	21	Dimer		−	d-Gal and d-Fuc	0.5	[17]
*Axinella polypoides II*	15	Monomer	−	−	d-Gal and d-Fuc	0.5	[17]
*Axinella corrugata I*	13.9	Hexamer	+	+	*N*,*N'*,*N''*-Triacetylchitotriose	N.D.	[18]
*Axinella corrugata II*	13.9	N.D	N.D.	+	*N*,*N'*,*N''*-Triacetylchitotriose	N.D.	[18]
*Cliona varians*	28.5	Tetramer	+	+	Galactose	N.D.	[21]
*Craniella australiensis*	17.8	Trimer	+	+	Asialo-PSM	27.6	[22]
*Cinachyrella alloclada*	17	Monomer/Dimer	+	+	Lactose	+	[23]
*Cinachyrella apion*	15.5	Octamer	N.D.	+	Lactose	N.D.	[20]
*Cinachyrella* sp.	16	Tetramer	N.D.	+	Lactose	N.D.	[24]
*Geodia cydonium*	13	Trimer	+	N.D.	Lactose	9.92	[19]
*Halichondria okadai*	30	Dimer	−	−	Galactose	N.D.	[12]
*Haliclona cratera*	29	Monomer	−	Relatively	α-1 Glycoprotein and Mucin	3.7	[25]
*Haliclona caerulea I*	14	Monomer	N.D.	−	N.D.	N.D.	[26]
*Haliclona caerulea II*	15	Dimer	N.D.	+	N.D.	N.D.	[26]
*Pellina semitubulosa*	34	Hexamer	N.D.	Relatively	Lactose	3.4	[27]

−, Absence; +, presence; N.D., not determined.

In most studies carried out using this material, bioactive proteins stand out for their notorious ability to perform biological functions [13], which will be the subject of the discussion throughout this review article. Generally, these lectins show great variability in the number of subunits from monomers [17,25,26] to octamers [20]. Despite this remarkable feature, lectins from marine sponge subunits have molecular weight values (Table 1) ranging from13.9 [18] to 34 kDa [27].

According to the literature, numerous lectins present in marine sponges have the characteristic of remaining active, even at high temperatures. Part of them are characterized by having hemagglutinating activity up to 70 °C, and other studies suggest that at 100 °C, there is irreversible denaturation of heat-resistant lectins present in some species. This moderate heat resistance is not primarily due to the presence of disulfide bonds in their structures, since at least five of the species isolated to date contain these connections [18,19,21,22,23].

In relation to carbohydrate specificity, it was observed that many lectins isolated from marine sponges bind to galactose and lactose. However, these are not the only carbohydrates to which these lectins show specificity. Lectins from marine sponges differ widely with respect to their glycosylation pattern, ranging from 0.5% to 27.6% of their composition [17,22].

The most widely-known lectins from marine sponges are those belonging to the genus *Cinachyrella*: *C. alloclada*, *C. apion* and *Cinachyrella* sp. [20,23,24]. Galectin from *Cinachyrella* sp. (CchG-1) is the most well-studied lectin, being the only one with physicochemical characteristics and a three-dimensional structure. CchG-1 is presented as a homotetramer with monomers of 16 kDa. As with the other lectins of the genus *Cinachyrella*, it has high affinity for lactose and is very heat-stable, resisting temperatures up to 100 °C [24].

**Figure 1:**
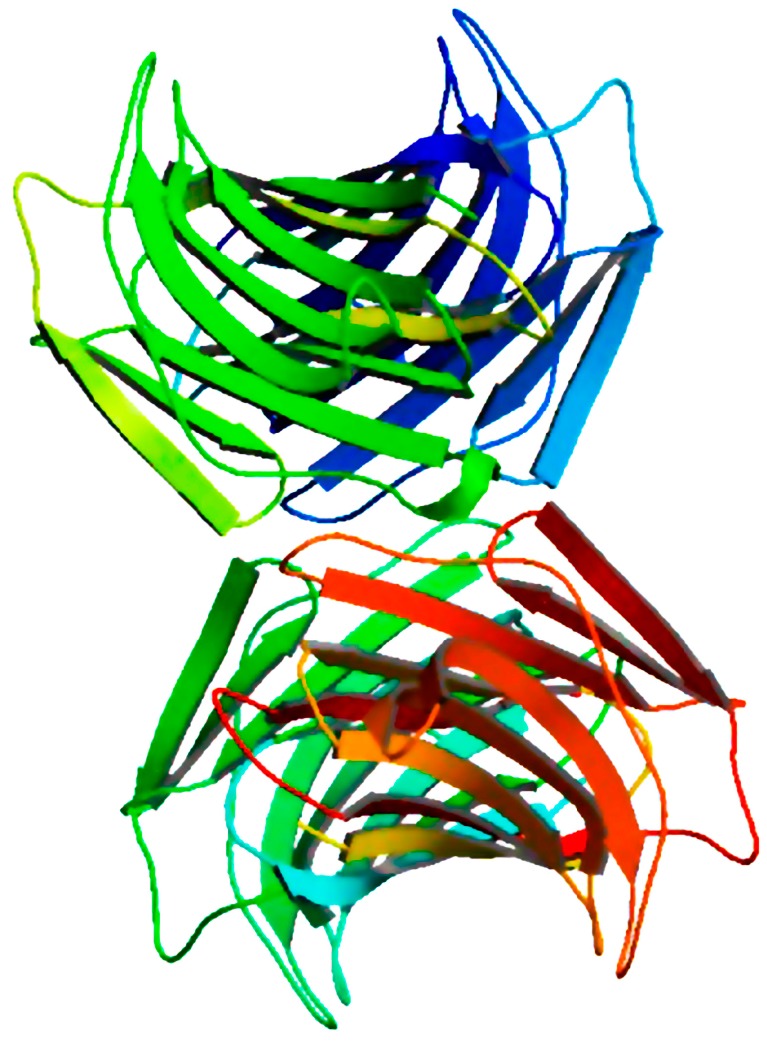
Tetrameric arrangement of *Cinachyrella* sp. galectin (CchG), the first three-dimensional structure of a lectin derived from a marine sponge (PDB Code: 4AGV, [28]).

Little has been studied about the structural features of lectins from marine sponges. CchG-1, a galectin isolated from *Cinachyrella* sp. (ball sponge), is the first three-dimensional structure of a sponge lectin determined by X-ray crystallography. The CchG-1 monomer is formed by two sequences of five antiparallel β-sheets paired face-to-face through a hydrophobic core. Therefore, CchG-1 consists of a rigid toroidal-shaped ‘donut-hole’ tetrameric arrangement (Figure 1), whose final structure is based on the presence of canonical dimers. This dimer of dimers interface is mediated by a novel structure that is stabilized by the packing of pairs of vicinal disulfide bonds between adjacent cysteines [28]. 

## 3. Biological Applications of Lectins from Marine Sponge 

**Figure 2:**
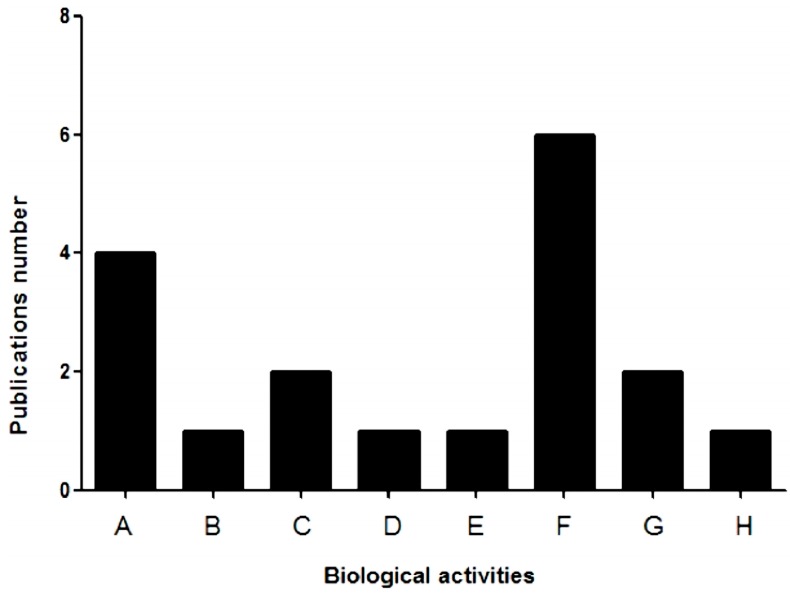
Biological activities found in lectins derived from marine sponges: (A) mitogenic activity; (B) binding to cells; (C) chemotaxis; (D) cytokine production; (E) pro-inflammatory; (F) toxicity; (G) recognition of *Leishmania*; (H) modulation of ion channels.

Whether eukaryotic or prokaryotic, all types of cells have carbohydrates and derivatives on their surface, as well as combinations of these with other macromolecules, such as oligosaccharides, glycoproteins or glycolipids. These macromolecules act as a potential connection point for lectins according to their affinity for this sugar. The binding of lectin to a sugar of the cell membrane leads to many cellular responses, many of which have an unknown molecular mechanism. However, and categorically, the binding of lectin to sugar is the key point in the definition of strategies for both the purification and determination of its biological activities [29]. All biological activities already published with lectins derived from marine sponges are quantitatively represented in Figure 2.

One of the most striking cellular responses induced by lectins is its ability to stimulate mitosis. Lectins from marine sponge, such as lectins from *Axinella corrugata* (ACL-1), *Craniella australiensis*, * Pellina semitubulosa* and *Cinachyrella alloclada*, are able to stimulate mitosis in mononuclear human cells, splenocytes and lymphocytes from mice and human lymphocytes, respectively [22,27,30].

In addition to mitogenic activity, ACL-1 can also be used as a tool for mapping tissue suspected of neoplasia. The results found by Dresch showed that this lectin complexed with biotin is able to recognize breast (T-47D, MCF7), colon (HT-29), lung (H460), ovarian (OVCAR-3) and bladder cancer cells (T24) by binding GalNAc and GlcNAc to the surface of these cells [31].

The recognition of other cell types, such as *Leishmania chagasi* promastigote forms, were found in lectin from *Cinachryrella apion* (CaL). Treatment with lectin against promastigotes of *Leishmania chagasi* was able to agglutinate the parasite; a dose of 1 mg/mL of lectin agglutinated 6.7 × 10^5^ cells. This agglutination does not occur in the presence of lactose, which is a fact that proves the specific binding of CaL to this sugar on the surface of the parasite, *L. chagasi* [20]. Another lectin with a similar property is CvL, a lectin from marine sponge *Cliona varians*, which is able to agglutinate 10^6^ cells of *L. chagasi* at a dose of 1 µg/mL [21].

The binding of lectin to cells often triggers intracellular signals leading to cell death. Like other molecules derived from marine organisms, lectins from marine sponges were also studied for their toxicity against some types of cells of biomedical importance. Lectin from *Halichondria crater* is an example of a lectin having toxicity against FemX and HeLa cells [25]. Lectins from *Cliona varians* (CvL) and *Cinachyrella apion* (CaL) also have this property. The antitumor activity induced by CaL in HeLa cells is due to the activation (non-exclusive) of the intrinsic apoptosis pathway, acting in a caspase-dependent and -independent manner, by inducing mitochondrial membrane permeability and the release of cytochrome and other proteins related to cell death. Moreover, CvL has antitumor activity against K562 leukemic cells differently from that presented by CaL against HeLa cells, since in this, case it occurs regardless of caspase activation [13,32].

The toxicity of lectins against tumor cells has been studied in some ways; however, lectins from marine sponges also have similar effects against microorganisms. A lectin from *Aplysina lacunosa* is capable of inhibiting the growth of bacteria *Enterococcus faecalis*, *Bacillus cereus*, *Escherichia coli* and *Salmonella enteritidis* [33]. The CvL is also able to inhibit the growth of bacteria *B. subtilis* and *S. aureus* [21].

Studies on plant lectins have shown that they exhibit an anti-inflammatory effect [34]; however, this property has not been found for lectins from marine sponges; instead, a pro-inflammatory effect was observed for CvL [35]. This lectin is also able to induce leukocyte migration in acute and chronic stages, but in the presence of galactose, fructose and sucrose, this migration is interrupted [35]. ACL-1 is also capable of stimulating neutrophil migration; however, in the presence of GlcNAc, chemotaxis is inhibited [18]. The production of interleukins 1 and 2 (IL-1 and IL-2) has been reported for lectin from the marine sponge *Pellina semitubulosa*, and these interleukins are correlated with inflammation [27].

Studies on neuroactive molecules have shown that galectin from marine sponge *Cinachyrella* sp. (CchG-1) is capable of modulating the glutamate receptor of rats, as the crude extract of this marine sponge was able to cause convulsions in animal models. The crude extract was able to slowly desensitize the ionotropic glutamate receptor (iGluR), proving to be inefficient by directly activating AMPA or kainate receptors in the absence of glutamate, which seems to act as a positive allosteric receptor. It was also observed that the equilibrium current of iGluR is irreversible. Thus, it is suggested that when connecting to iGluR, lectin CchG-1 plays a role similar to an excitatory neurotransmitter in the central nervous system (CNS). However, the authors did not rule out the possibility that there is still an indirect modulation capacity of AMPA or kainite receptors [24]. 

## 4. Conclusions

Marine sponges are a promising source of bioactive lectins, which are structurally diverse, many of them in the form of glycoproteins. They often have properties that allow withstanding high temperatures. Many lectins from marine sponge bind to the galactose, lactose or oligosaccharides of β-linked galactose, but not always exclusively to these carbohydrates. These proteins have great potential for biotechnological applications, shown by activities ranging from the ability to modulate ion channels to toxicity against bacteria and tumor cells. Currently, toxicity against microorganisms and tumor cells are activities of great relevance provided by these proteins from marine sponges. These lectins can be used as a possible drug against microorganisms, due to their antibiotic activity or against tumor cells. However, further studies on this subject need to be conducted with the purpose of investigating their mechanisms of action and whether these proteins also show no toxicity to other types of cells that should not suffer from this action.
